# Pathogenic and transcriptomic differences among porcine reproductive and respiratory syndrome viruses from distinct lineages in piglets

**DOI:** 10.1186/s13567-025-01659-w

**Published:** 2025-11-28

**Authors:** Linxing Tian, Xingran Wang, Zhenxiang Rong, Mengxin Zhang, Yawei Sun, Ruiqu Zhou, Jiahui Ma, Chao Zhang, Shudan Liu, Nan Cao, Zihui Hu, Jiyuan Luo, Xiangmin Li, Ping Qian

**Affiliations:** 1 National Key Laboratory of Agricultural Microbiology, Hubei Hongshan Laboratory, Huazhong Agricultural University, Wuhan, 430070 Hubei China; 2 College of Veterinary Medicine, Huazhong Agricultural University, Wuhan, 430070 Hubei China; 3 Hubei Jiangxia Laboratory, Wuhan, 430200 Hubei China; 4 Key Laboratory of Preventive Veterinary Medicine in Hubei Province, The Cooperative Innovation Center for Sustainable Pig Production, Wuhan, 430070 Hubei China

**Keywords:** Porcine reproductive and respiratory syndrome virus, recombination, pathogenicity, transcriptomic analysis, host responses

## Abstract

Porcine reproductive and respiratory syndrome virus (PRRSV) is a highly recombinogenic pathogen that threatens global swine production. Multiple PRRSV lineages co-circulate in China, with lineage 1.8 being the predominant epidemic strain. However, the pathogenic characteristics and differences among strains from distinct lineages remain insufficiently studied. In this study, three novel recombinant PRRSV strains (GX-2428, GX-3264, and GX-5430) were isolated in Guangxi, China. Phylogenetic analysis of the ORF5 gene classified the three strains into lineage 3 (QYYZ-like), lineage 1.8 (NADC30-like), and lineage 1.5 (NADC34-like), respectively. Pathogenicity tests in piglets demonstrated that, compared with the control group, both GX-2428 and GX-3264 induced significant fever, whereas GX-5430 caused only a transient and milder febrile response. Infected piglets exhibited elevated levels of pro-inflammatory (IL-1β and TNF-α) and immunomodulatory cytokines (IL-4 and IL-10) compared to the control group. Postmortem analysis revealed that although viral shedding had ceased, high viral loads persisted in the lungs, tonsils, and lymph nodes of the infected piglets. Transcriptomic analysis of piglet lung tissues revealed that GX-5430 infection predominantly enriched pathways related to cellular transformation, signal transduction, and metabolic reprogramming. However, infections with GX-2428 or GX-3264 were significantly enriched in immune-related pathways, thereby inducing stronger immune activation and inflammatory responses. In conclusion, these findings highlight the recombination characteristics and lineage-specific pathogenic mechanisms of PRRSV, providing novel insights for the development of future prevention and control strategies.

**Supplementary Information:**

The online version contains supplementary material available at 10.1186/s13567-025-01659-w.

## Introduction

Porcine reproductive and respiratory syndrome (PRRS), caused by the PRRSV [1], is a widespread disease characterized by high fever, acute reproductive disorders in sows, and respiratory symptoms in pigs of all ages [2]. PRRSV is an enveloped, positive-sense single-stranded RNA virus that belongs to the genus *Betaarterivirus*, family *Arteriviridae*, and order *Nidovirales* [3]. The PRRSV genome is approximately 15.4 kb in length and encodes 16 nonstructural proteins (NSP1α, NSP1β, NSP2, NSP2TF, NSP2N, NSP3-NSP6, NSP7α, NSP7β, and NSP8-NSP12) and 8 structural proteins (GP2a/ORF2a, E/ORF2b, GP3/ORF3, GP4/ORF4, GP5/ORF5, 5a/ORF5a, M/ORF6, and N/ORF7) [4–6]. PRRSV is genetically diverse and can be classified into two distinct genotypes: the European genotype (type 1) and the North American genotype (type 2), which differ from each other by approximately 40% across their genomes [7]. In China, PRRSV-2 is the predominant circulating genotype and is classified into nine distinct lineages based on the phylogenetic analysis of the ORF5 gene [8].

In 2006, a highly pathogenic PRRSV (HP-PRRSV) belonging to lineage 8 emerged in China and caused significant economic losses to the Chinese pig industry due to its high mortality rates [9]. In 2010, QYYZ and GM2 (lineage 3) were reported to be widespread in southern China [10]. In 2013, the NADC30-like strain belonging to lineage 1.8 emerged and spread widely across China [11]. This strain exhibits moderate virulence but shows a high propensity for recombination with other PRRSV strains, leading to the emergence of new variant strains [12]. In 2018, a new PRRSV strain, designated as the NADC34-like strain and classified into lineage 1.5, was reported in China. The detection rate of this strain has increased annually [13]. Due to its high mutation and recombination rates, PRRSV has led to the emergence of various new recombinant strains [14]. These recombinant strains display unique epidemiological characteristics, posing significant challenges for disease prevention and control in pig farming [15, 16]. However, studies on the pathogenicity of recombinant PRRSV strains from different lineages remain limited.

Compared with traditional approaches such as qPCR and protein-based assays, which are well suited for targeted validation of known genes or proteins, transcriptome sequencing provides a comprehensive and unbiased assessment of host responses. It enables the discovery of novel differentially expressed genes (DEGs) and the enrichment of functional pathways under specific conditions and offering valuable insights into virus–host interactions [17]. The host innate immune response plays a critical role in defending against PRRSV infection, and different PRRSV strains can elicit distinct immune profiles [18, 19]. The low-virulence SD53 strain activates innate immune pathways such as the “Toll-like receptor signaling pathway” and “NOD-like receptor signaling pathway”. In contrast, the highly virulent HuN4 strain suppresses pathways associated with adaptive and regulatory immune responses, including “Th1 and Th2 cell differentiation” and the “TGF-beta signaling pathway” [20]. Additionally, the lineage 1.5 strain YC-2020 upregulates multiple proinflammatory cytokines, albeit to a lesser extent than the lineage 8 strain JXA1 [21], indicating that PRRSV-induced immune responses are dependent on viral lineage.

In this study, we analyzed the genomic characteristics of three isolated PRRSV strains (GX-2428, GX-3264, and GX-5430) and evaluated their pathogenicity in piglets. Furthermore, the underlying mechanisms responsible for the differences in pathogenicity among the strains were investigated through transcriptomic analysis. By comparing the pathogenic differences among recombinant strains from different lineages, this study aims to deepen our understanding of PRRSV and offer novel insights for its prevention and control.

## Materials and methods

### Sample collection and processing

In 2024, serum and lung tissue samples suspected of PRRSV infection were collected from multiple pig farms in Guangxi, China. After three freeze–thaw cycles, the samples were stored at -80°C for subsequent analysis.

### Virus isolation and identification

For virus isolation, the positive tissue was filtered through 0.22-µm filters and inoculated onto porcine alveolar macrophages (PAMs). The cells were cultured in Roswell Park Memorial Institute (RPMI) 1640 medium (Gibco, Waltham, MA, USA) supplemented with 10% fetal bovine serum at 37 °C in a 5% CO₂ humidified atmosphere. When approximately 80% of the virus-infected cells exhibited cytopathic effect (CPE), the virus was harvested through freeze–thaw cycles. The isolated strains were identified by indirect immunofluorescence assay (IFA) as previously described [22]. IFA experiments were performed using PRRSV N protein antibody (GeneTex, Inc., Irvine, CA, USA) and Alexa Fluor 488 goat anti-rabbit IgG antibody (Abcam, Cambridge, UK).

### Genome sequencing, genetic evolution analysis, and recombination analysis

PRRSV was sequentially passaged on PAMs, and the third passage was used for whole-genome sequencing. The phylogenetic tree was constructed using the maximum likelihood method with 1,000 bootstrap replicates in MEGA 7 (Molecular Evolutionary Genetics Analysis Version 7.0) to evaluate genetic evolution [23]. Seven methods (RDP, Bootscan, GENECONV, Chimaera, MaxChi, SiScan, and 3Seq) were employed using the recombination detection program RDP 4.10 (Recombination Detection Program Version 4.10) to evaluate recombination events [24]. A recombination event was considered identified when at least five of the seven methods detected recombination signals in RDP 4.10. Furthermore, SimPlot version 3.5.1 was used to further investigate the recombination events identified by RDP 4.10, with a window width of 200 base pairs (bp) and a step size of 20 bp to identify potential parental strains [25].

### Pathogenicity studies in piglets

To evaluate the pathogenicity of the three isolated PRRSV strains, twenty 5-week-old piglets free of African swine fever virus (ASFV), classical swine fever virus (CSFV), pseudorabies virus (PRV), porcine circovirus type 2 (PCV2), and PRRSV were randomly divided into four groups: GX-2428 group (*n* = 5), GX-3264 group (*n* = 5), GX-5430 group (*n* = 5), and control group (*n* = 5). Piglets in the infection groups were inoculated with 4 × 10^5^ TCID₅₀ of their respective strains via a combination of intranasal (2 mL) and intramuscular (2 mL) routes. Control group piglets received an equal volume of RPMI 1640 medium via the same routes. After inoculation, the rectal temperatures of the piglets were monitored daily, and body weights were recorded at 0, 7, 14, and 21 days post-infection (dpi). Serum, nasal swabs, and fecal swabs were collected from piglets at 0, 3, 5, 7, 10, 14, and 21 dpi for subsequent analyses. Viral copy numbers were determined by RT-qPCR using ORF6 primers and a probe (Additional file 1) to monitor viral shedding and clearance. The standard curve was generated by performing tenfold serial dilutions of a plasmid containing the PRRSV ORF6 fragment. Ct values obtained from RT-qPCR were then fitted to this standard curve to calculate the viral genome copy numbers in each sample. At 21 dpi, all piglets were euthanized for necropsy, and tissue samples were collected for further analysis.

### Library preparation and transcriptome sequencing

Total RNA was extracted from lung tissues using TRIzol reagent according to the manufacturer’s instructions. High-quality RNA samples were submitted to MetWare Biotechnology (Wuhan, China) for library preparation and RNA-sequencing (RNA-Seq). Raw sequencing data underwent quality control and filtering to obtain clean reads, which were then aligned to the *Sus scrofa* reference genome using HISAT2 [26, 27].

### Statistical analysis

Statistical analyses were performed using GraphPad Prism 8.0.2 (GraphPad Software Version 8.0.2, Inc., San Diego, CA, USA), with data presented as mean ± standard deviation (SD). Comparisons were performed through one-way or two-way ANOVA followed by Tukey’s test. A *p* value of < 0.05 was considered to indicate statistical significance.

## Results

### Virus isolation and phylogenetic analysis

**Figure 1:**
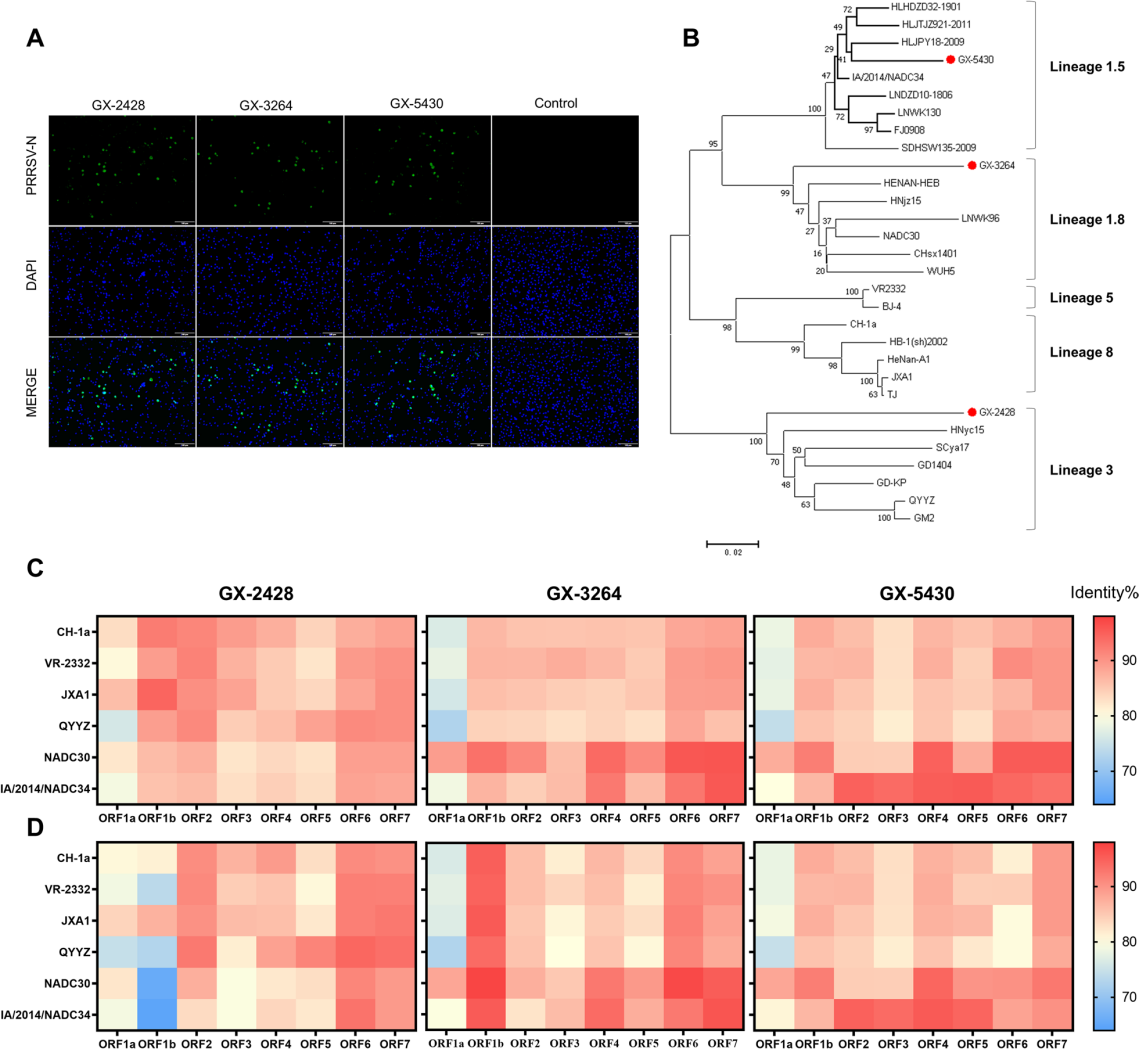
**Virus isolation and phylogenetic analysis. A** Indirect immunofluorescence assay (IFA) was used to detect the PRRSV strains in infected PAMs. **B** A phylogenetic tree was constructed based on ORF5 gene sequences of the GX-2428, GX-3264, GX-5430, and reference strains. **C** Nucleotide sequence identity between GX-2428, GX-3264, GX-5430, and six PRRSV reference strains. **D** Amino acid sequence identity of ORF5 among the same strains.

PRRSV was isolated by inoculating diseased tissue samples onto PAMs, and viral replication was confirmed by IFA (Figure 1A). The complete genome lengths of GX-2428 (GenBank accession number: PV240233), GX-3264 (GenBank accession number: PV240231), and GX-5430 (GenBank accession number: PV240232) were 14 993, 15 013, and 15 019 nucleotides (nt), respectively, excluding the poly(A) tails at the 3′ end. A phylogenetic tree based on the ORF5 gene was constructed, revealing that GX-2428 belongs to lineage 3, GX-3264 to lineage 1.8, and GX-5430 to lineage 1.5 (Figure 1B). The genomic characteristics of these strains were further evaluated by comparing their nucleotide and amino acid sequences with those of reference strains. Genome similarity analysis revealed that GX-2428 presented greater sequence identity with the JXA1-like strain in the nonstructural protein (NSP) region (ORF1a and ORF1b) and with the QYYZ-like strain in the structural protein (SP) region (ORF2–ORF7). GX-3264 demonstrated higher overall similarity to the NADC30-like strain, whereas GX-5430 exhibited higher sequence identity with the NADC30-like strain in the NSP region and with the NADC34-like strain in the SP region (Figures 1C and D).

**Figure 2:**
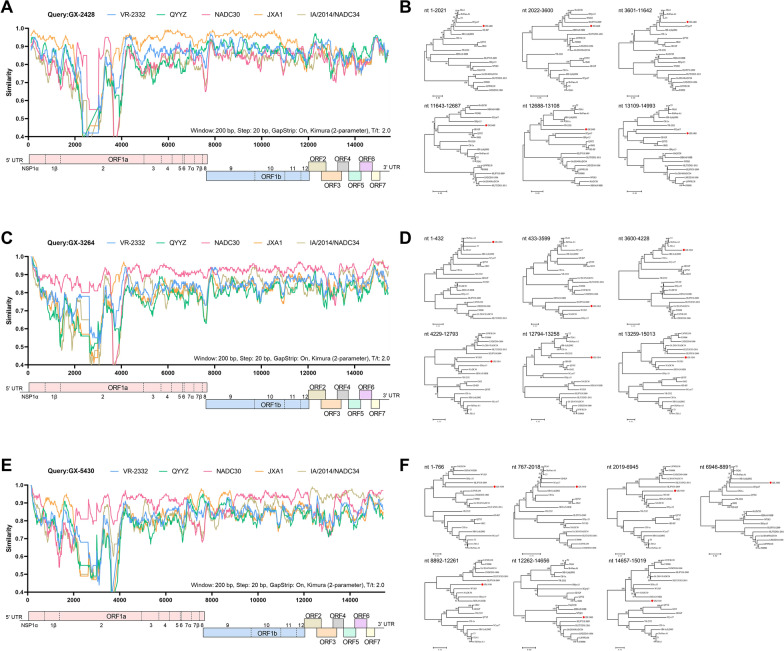
**Recombination analysis of PRRSV strains. A**, **C**, and **E** Recombination events in GX-2428, GX-3264, and GX-5430 were analyzed using RDP4 and SimPlot by comparison with representative reference strains. **B**, **D**, and **F** Phylogenetic trees were constructed based on different recombination regions of each PRRSV strain. The target strains (GX-2428, GX-3264, and GX-5430) were labeled with red circles.

Recombination analysis revealed multiple recombination events in GX-2428, GX-3264, and GX-5430, indicating that all three PRRSV strains are recombinant viruses. Five potential recombination breakpoints were identified in the complete genome of GX-2428, located in NSP2 (nt 2022 and 3601), NSP12 (nt 11 643), ORF3 (nt 12 688), and ORF4 (nt 13 109) regions (Figure 2A). These breakpoints delineated six recombination regions, with the JXA1-like strain serving as the major parental strain (regions nt 1–2021 and 3601–11 642), and the NADC30-like (nt 2022–3600), QYYZ-like (nt 11 643–12 687 and 13 109–14 993), and VR-2332-like (nt 12 688–13 108) strains served as minor parental strains (Figure 2B). For GX-3264, five recombination breakpoints were identified across the complete genome, located in the NSP1 (nt 433), NSP2 (nt 3600 and 4229), ORF3 (nt 12 794), and ORF4 (nt 13 259) regions (Figure 2C). These breakpoints defined six distinct recombination regions, with the NADC30-like strain serving as the major parental strain (nt 433–3599, 4229–12 793, and 13 259–15 013), and the JXA1-like strain (nt 1–432 and 3600–4228) and the VR-2332-like strain (nt 12 794–13 258) served as minor parental strains (Figure 2D). In GX-5430, six recombination breakpoints were identified in the NSP1 (nt 767), NSP2 (nt 2019 and 6946), NSP9 (nt 8892), ORF2 (nt 12 262), and ORF7 (nt 14 657) regions (Figure 2E). These breakpoints defined seven recombination regions, with the NADC30-like strain serving as the major parental strain (nt 1–766, 2019–6945, 8892–12 261, and 14 657–15 019), and the JXA1-like strain (nt 767–2018 and 6946–8891) and the NADC34-like strain (nt 12 262–14 656) served as minor parental strains (Figure 2F). All three strains underwent recombination events in the NSP2 region, which was partially or completely replaced by the corresponding region from the NADC30-like strain, suggesting that recombination involving the NSP2 region of NADC30-like strains contributes to PRRSV immune evasion.

### Clinical symptoms in piglets

**Figure 3:**
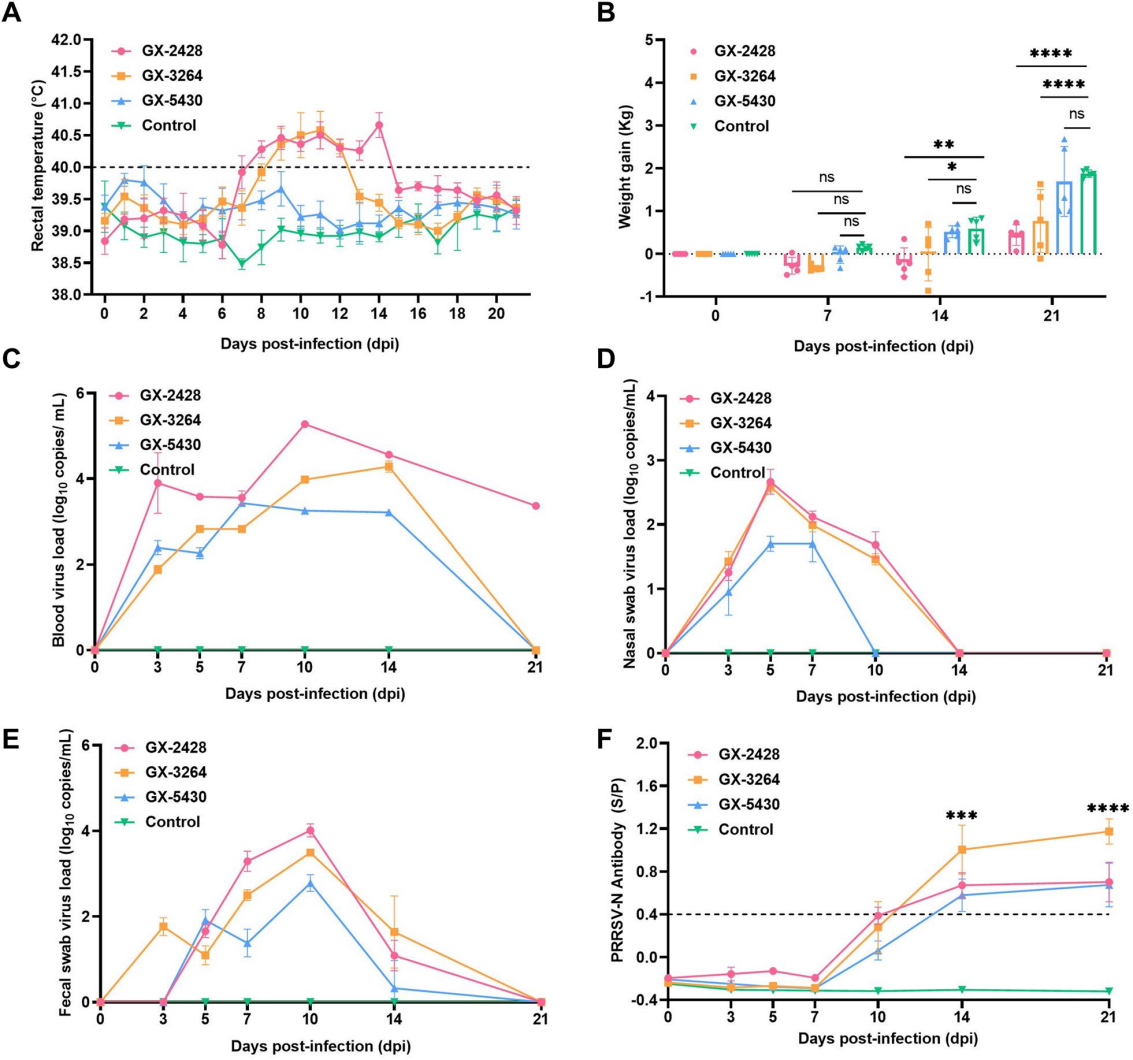
**Comprehensive assessment of piglets in different infection groups. A** Changes in the rectal temperature of piglets in each group after challenge, with the fever cut-off value set at 40.0 ℃. **B** Weight gain of piglets during the experimental period. **C**–**E** Viral load detection in blood, nasal swabs, and fecal swabs at the indicated time points. **F** PRRSV-specific N protein antibody levels were measured by ELISA. An S/P (sample OD–negative OD)/(positive OD–negative OD) ratio ≥ 0.4 was considered positive for anti-PRRSV antibodies. Data are presented as mean ± SD. *p < 0.05; **p < 0.01; ***p < 0.001; ****p < 0.0001; ns, no significant difference.

Five-week-old piglets were used to evaluate the pathogenicity of the GX-2428, GX-3264, and GX-5430 strains. Piglets infected with GX-2428 or GX-3264 exhibited significant fever symptoms. In contrast, piglets infected with the GX-5430 strain did not exhibit a significant elevation in body temperature compared to the control group (Figure 3A). The weight gain was significantly lower in the GX-2428 and GX-3264 groups than in the control group (*p* < 0.0001), whereas the GX-5430 group exhibited only a slight, non-significant reduction (*p* > 0.05; Figure 3B).

### Detection of virus and humoral immune response

Nasal swabs, fecal swabs, and blood samples were collected from each group at 0, 3, 5, 7, 10, 14, and 21 dpi to monitor viral shedding and PRRSV N protein-specific antibody levels. Viral RNA was detected in both nasal and fecal swabs of GX-3264-infected piglets at 3 dpi, indicating the early onset of viral shedding. In contrast, viral shedding in piglets infected with GX-2428 or GX-5430 was first detected in nasal swabs at 3 dpi and in fecal swabs at 5 dpi, suggesting that GX-3264 induces more rapid viral shedding kinetics than the other two strains (Figures 3D and E). The GX-2428 group exhibited peak viremia at 10 dpi (10^5.27^ copies/mL), which gradually decreased to 10^3.37^ copies/mL by 21 dpi. In comparison, the GX-3264 and GX-5430 groups reached peak viremia at 14 dpi (10^4.28^ copies/mL) and 7 dpi (10^3.43^ copies/mL), respectively, with viral loads becoming undetectable in both groups by 21 dpi. Throughout the experiment, viremia levels in the GX-2428 group remained consistently higher than those observed in the GX-3264 and GX-5430 groups, indicating that GX-2428 replicates more efficiently in piglets (Figure 3C). The serum levels of PRRSV N protein-specific antibodies were detected using an enzyme-linked immunosorbent assay (ELISA) kit. In all infected groups, the antibody levels against the N protein were mildly induced at 10 dpi and gradually increased thereafter (Figure 3F).

### Histopathological lesions and virus distribution

**Figure 4:**
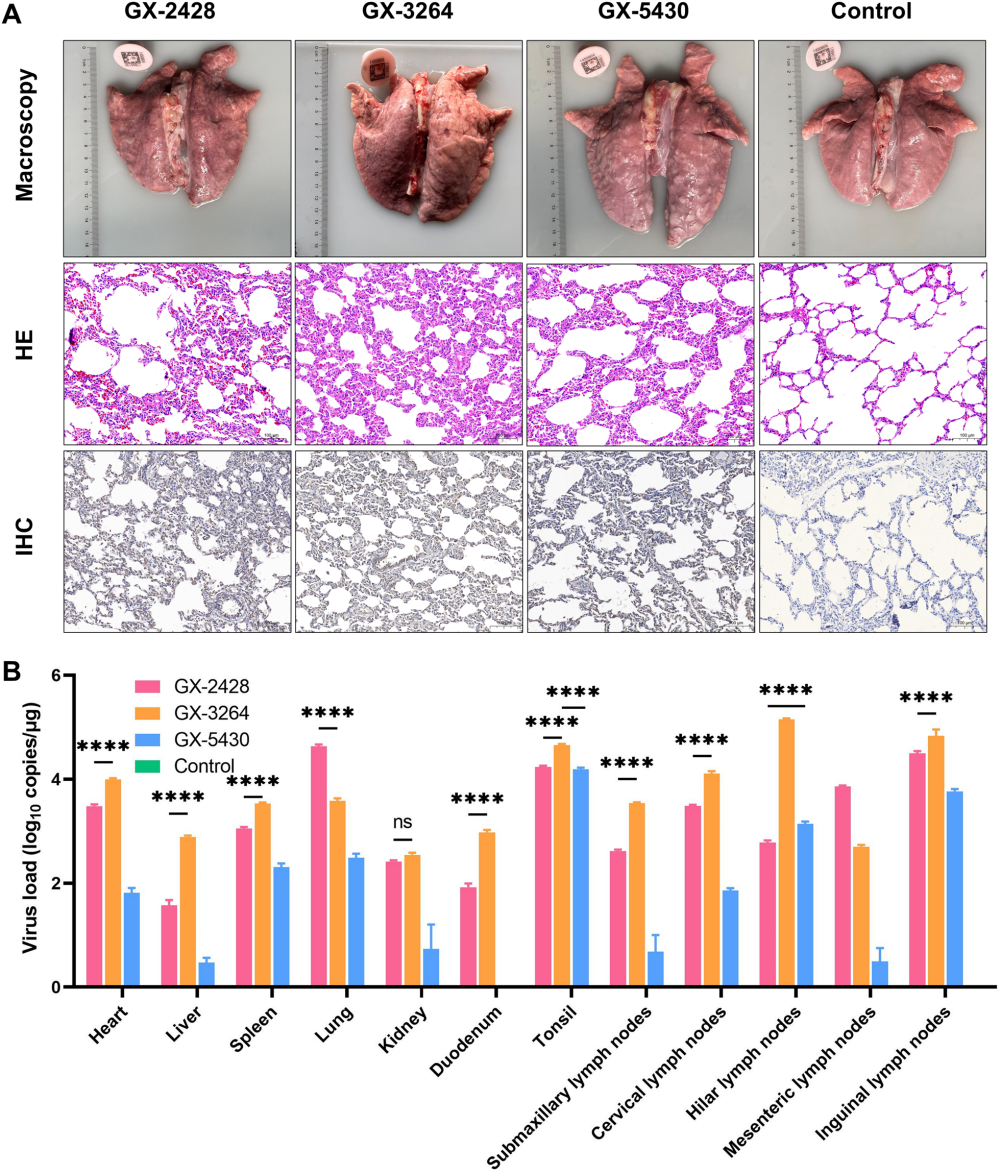
**Lung pathology and tissue viral load analysis. A** Gross and histological lesions were observed in the lungs of piglets infected with GX-2428, GX-3264, or GX-5430. **B** Viral load in various tissues of piglets from each group, as determined by RT‒qPCR. Data are presented as mean ± SD. ****p < 0.0001; ns, no significant difference.

All piglets were euthanized at 21 dpi to evaluate histopathological lesions and viral distribution in multiple tissues. At necropsy, piglets in the infected groups exhibited interstitial pneumonia, characterized by pulmonary consolidation and hemorrhagic spots. Compared with the GX-3264 and GX-5430 groups, the GX-2428 group exhibited more severe pathological lesions. Histopathological analysis demonstrated pronounced alveolar septal thickening, extensive lymphocytic infiltration, and architectural disruption in the lungs of piglets infected with GX-2428 or GX-3264. In contrast, lungs from GX-5430-infected piglets showed only mild histopathological alterations, whereas no significant lesions were observed in the control group. Immunohistochemistry (IHC) using PRRSV N protein-specific antibodies confirmed the presence of positive PRRSV signals in the lung tissues of infected piglets, whereas no such signals were detected in the control group (Figure 4A). Viral distribution was assessed in various piglet tissues at 21 dpi. In the GX-2428 group, the highest viral load was detected in the lungs, followed by the inguinal lymph nodes and tonsils. In contrast, in piglets infected with GX-3264 or GX-5430, the virus was predominantly localized in immune-related organs, including the tonsils, hilar lymph nodes, and inguinal lymph nodes (Figure 4B). Compared with GX-2428 and GX-3264, piglets infected with GX-5430 exhibited milder lung lesions and slightly lower viral loads across multiple tissues, indicating that GX-5430 was a less pathogenic strain.

### Serum cytokine levels

**Figure 5:**
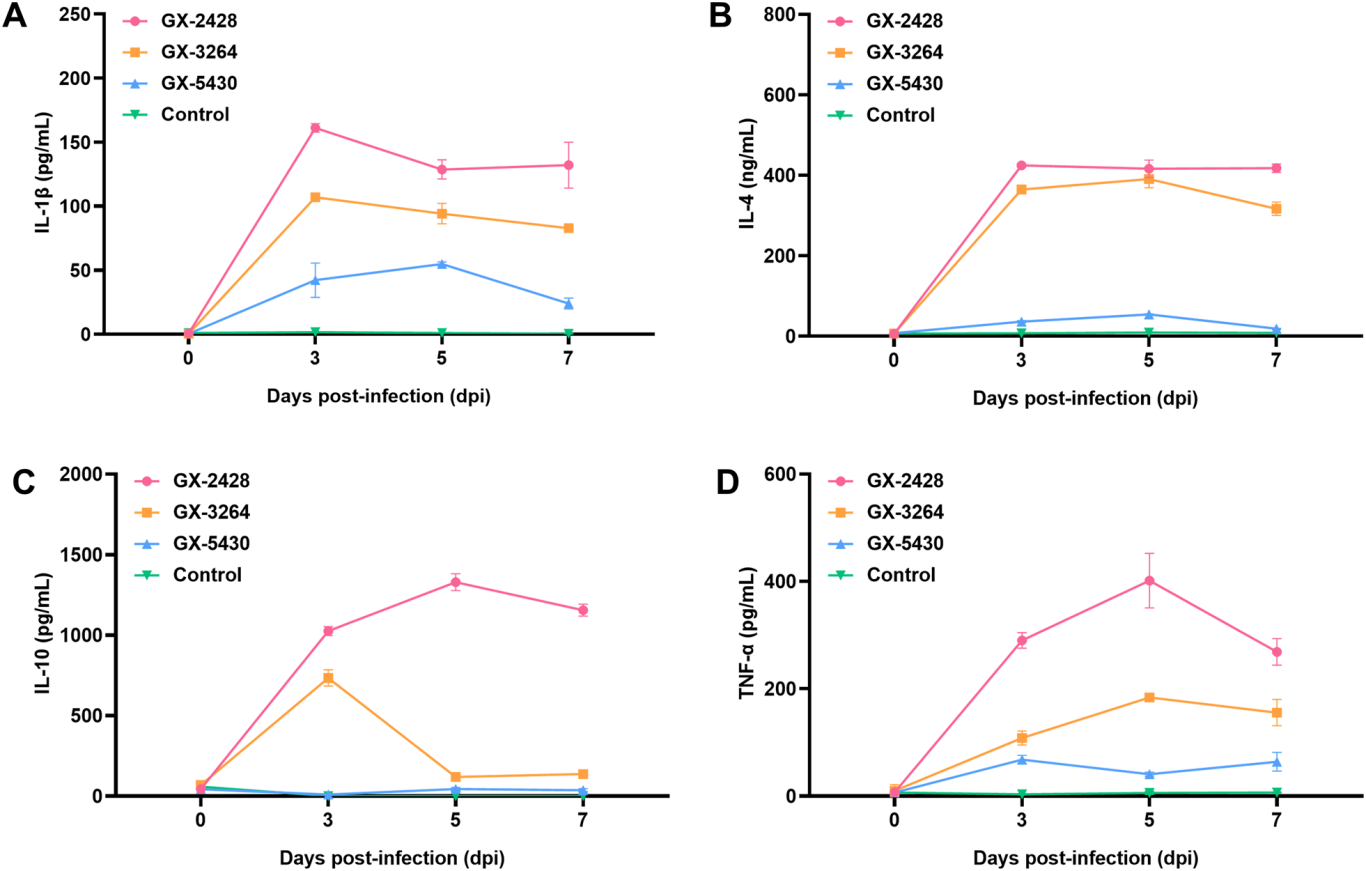
**Cytokine profiles in PRRSV-infected piglets. A**–**D** Serum levels of IL-4 (**A**), IL-10 (**B**), IL-1β (**C**), and TNF-α (**D**) in PRRSV-infected piglets were measured using commercial ELISA kits. Data are presented as mean ± SD.

To investigate the effects of the three PRRSV strains on cytokine secretion in piglets, serum levels of IL-1β, IL-4, IL-10, and TNF-α were measured using commercial ELISA kits (Neobioscience, China). The levels of the inflammatory cytokines IL-1β and TNF-α initially increased but then decreased in all PRRSV-infected groups, with the GX-2428 group exhibiting the highest levels (Figures 5A and D). In the GX-5430 group, the IL-4 levels initially increased, peaked at 5 dpi, and then decreased, remaining significantly lower than those in the GX-2428 and GX-3264 groups (Figure 5B). The immunomodulatory cytokine IL-10 was significantly elevated in both the GX-2428 and GX-3264 groups, peaking at 5 dpi and 3 dpi, respectively (Figure 5C). In summary, GX-2428 and GX-3264 induce excessive inflammatory responses in piglets.

### Transcriptomic analysis of piglets infected with GX-2428, GX-3264, and GX-5430 PRRSV strains

**Figure 6:**
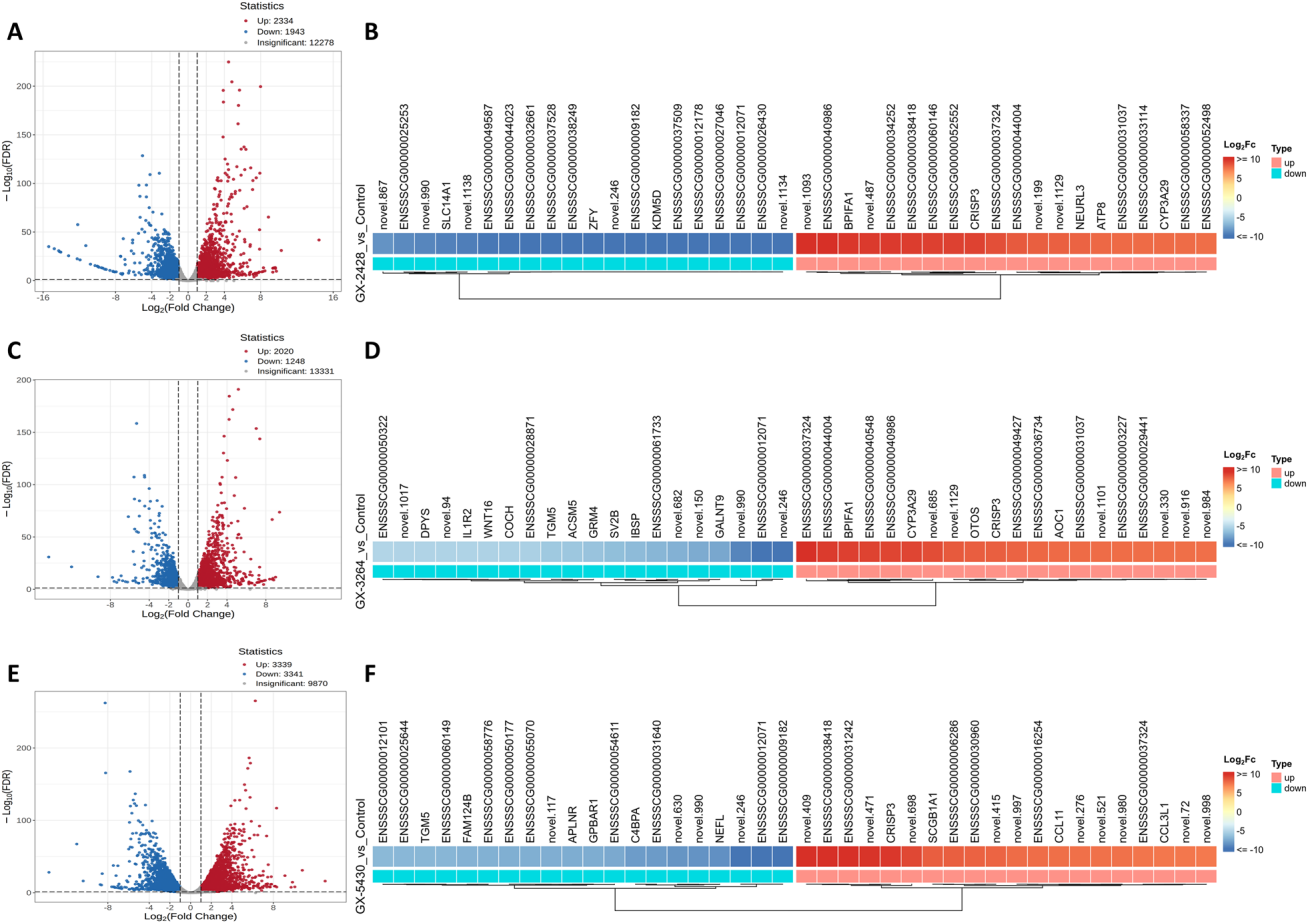
**DEGs analysis between PRRSV-infected groups and control group.** Volcano plots (**A**, **C**, and **E**) showing the DEGs in the lung tissues of piglets infected with GX-2428, GX-3264, and GX-5430, respectively, compared with those in the control group. DEGs were identified based on the criteria of | log₂ (Fold Change) |≥ 1 and a false discovery rate (FDR) < 0.05. Red dots indicate significantly upregulated genes, blue dots indicate significantly downregulated genes, and gray dots indicate nonsignificant genes. Heatmaps (**B**, **D**, and **F**) of the top 20 upregulated and downregulated DEGs (sorted by log₂ (Fold Change)) in each PRRSV-infected group.

To elucidate the impact of different PRRSV lineage strains on the lungs of infected piglets, transcriptomic analysis was performed on lung tissues collected at 21 dpi. Principal component analysis (PCA) demonstrated high intragroup consistency and distinct intergroup separation, indicating the reliability of the transcriptomic data (Additional file 2A). Differential gene expression analysis identified 4277 differentially expressed genes (DEGs) in the lungs of GX-2428-infected piglets, including 2334 upregulated and 1943 downregulated genes. In the GX-3264-infected group, 3268 DEGs were identified, including 2020 upregulated and 1248 downregulated genes. Notably, GX-5430 infection led to the identification of 6680 DEGs, with 3339 genes upregulated and 3341 genes downregulated (Figures 6A, C, and E). Further analysis revealed that genes such as *BPIFA1*, *CRISP3*, *IRF7*, *STAT1*, *CXCL10*, *IFIT3*, *ISG15*, *RSAD2*, *TYK2*, *GPBAR1*, and *TGM5* were significantly upregulated in infected groups. These genes are involved in immune regulation, antiviral defense, and activation of inflammatory signaling pathways. In contrast, genes such as *IL1R2*, *GRM4*, *DPYS*, *WNT16*, *GALNT9*, *SV2B*, *IBSP*, and *COCH*, which are associated with neural function, metabolic regulation, and signal transduction, were significantly downregulated (Figures 6B, D, and F). These results suggest that PRRSV infection induces a complex and strain-specific transcriptional response, potentially involving interactive regulation between immune activation and disruption of tissue homeostasis.

**Figure 7:**
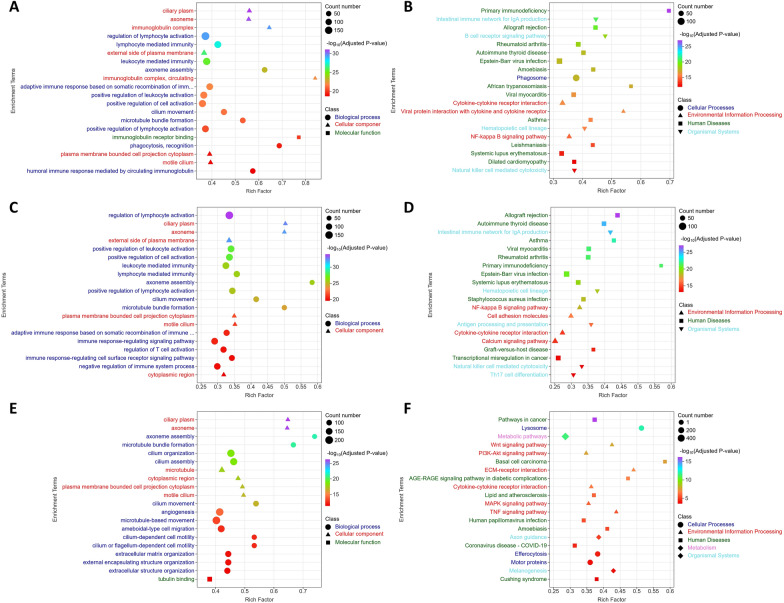
**Functional annotation analysis of DEGs between PRRSV-infected groups and control groups. A** and **B** GO and KEGG enrichment analyses of DEGs in lung tissues from the GX-2428 group. **C** and **D** GO and KEGG enrichment analyses of DEGs in lung tissues from the GX-3264 group. **E** and **F** GO and KEGG enrichment analyses of DEGs in lung tissues from the GX-5430 group.

Gene Ontology (GO) classification analysis revealed that the number of upregulated genes slightly exceeded that of downregulated genes in the GX-2428 and GX-3264 groups. In contrast, the GX-5430 group exhibited a higher number of downregulated genes (Additional files 2B–D), suggesting that this strain triggered a more pronounced suppressive host response. GO enrichment analysis revealed that GX-5430 infection was significantly enriched in biological processes such as “angiogenesis,” “extracellular matrix organization,” and “cilium-dependent cell motility” (Figure 7E). Conversely, infections with GX-2428 or GX-3264 primarily activated immune-related biological processes, including “regulation of lymphocyte activation,” “positive regulation of cell activation,” and “lymphocyte-mediated immunity,” indicating that these two strains induced robust adaptive immune responses (Figures 7A and C).

The Kyoto Encyclopedia of Genes and Genomes (KEGG) enrichment results exhibited a trend similar to that of the GO results. GX-5430 infection was associated with weaker enrichment of immune-related pathways and predominantly involved host pathways related to cancer, signal transduction, and metabolic reprogramming (Figure 7F). In comparison, GX-3264 infection induced moderate immune activation, which involved mainly immune regulatory pathways and helper T cell-related responses, such as “Th17 cell differentiation,” “antigen processing and presentation,” and “calcium signaling pathway” (Figure 7D). Compared with the other two strains, GX-2428 infection led to significant enrichment of classical immune-related pathways, including the “B cell receptor signaling pathway,” “natural killer cell-mediated cytotoxicity,” and “cytokine–cytokine receptor interaction” (Figure 7B). These intense immune responses trigger severe inflammation, resulting in tissue damage and fever symptoms.

**Figure 8:**
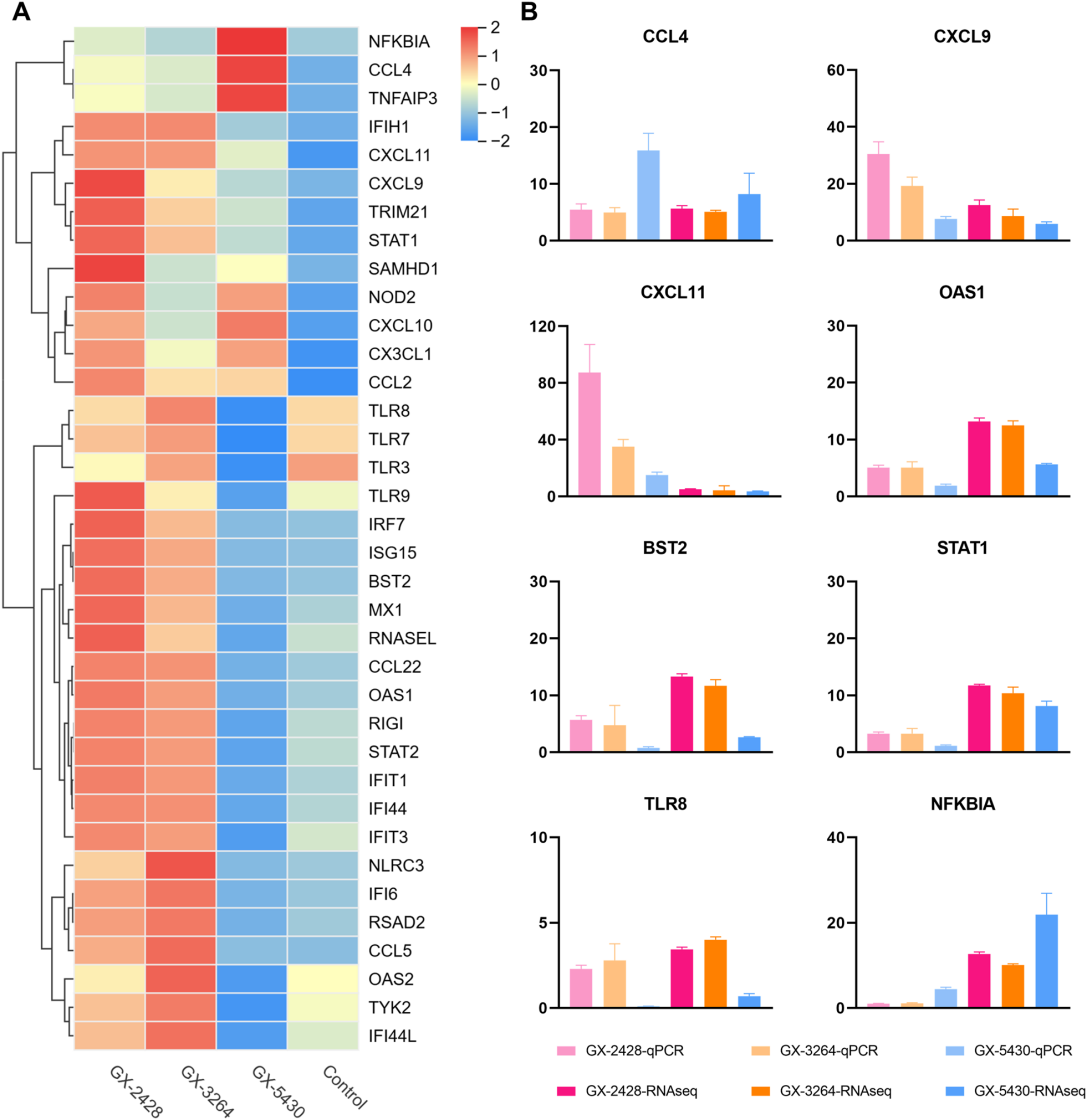
**Analysis of the expression of innate immunity-related genes. A** Heatmap of the expression profiles of key innate immune-related genes. The horizontal axis represents different experimental groups, and the vertical axis represents selected genes. High expression levels are indicated in red, whereas low expression levels are shown in blue. **B** The expression levels of innate immunity-related genes were compared using FPKM data from RNA-seq and qPCR.

Multiple innate immunity-related genes, including pattern recognition receptors, interferon-stimulated genes, chemokines, key components of the JAK–STAT pathway, and antiviral effector genes, were broadly upregulated in the GX-2428 and GX-3264 groups. These findings suggest that GX-2428 and GX-3264 induce strong innate immune activation and inflammatory responses. In contrast, the expression of these innate immunity-related genes was significantly downregulated in the GX-5430-infected group, whereas negative regulators of the NF-κB signaling pathway were upregulated, indicating that GX-5430 suppresses immune-related pathways, thereby evading immune surveillance and inducing a weaker immune response (Figure 8A). The expression levels of eight key genes were validated by quantitative PCR, and the results were consistent with the transcriptomic data, further confirming the accuracy of the transcriptome data analysis (Figure 8B). These results further suggest that, compared with GX-2428 and GX-3264, GX-5430 induces a milder inflammatory response, which leads to less severe tissue damage.

## Discussion

Since its emergence, PRRSV has posed a major threat to the global swine industry since its emergence. In China, PRRSV-2 strains are primarily classified into four lineages: 1, 3, 5, and 8. Previous studies have demonstrated that PRRSV pathogenicity and virulence are closely associated with the ORF5 gene [28, 29], which also plays a critical role in eliciting host immune responses [30]. Therefore, phylogenetic analysis based on ORF5 sequences is crucial for elucidating the evolutionary dynamics and pathogenic mechanisms of PRRSV.

In this study, three recombinant PRRSV strains (GX-2428, GX-3264, and GX-5430) were isolated from pig farms in Guangxi, China. Recombination breakpoints were identified within the NSP regions, specifically in NSP1, NSP2, NSP9, and NSP12. Notably, NSP2 was a common recombination breakpoint across all three recombinant strains, suggesting that this region remains a critical site for recombination within the nonstructural protein-coding region [31, 32]. Recombination events in the NSP2 region often result in partial or complete replacement by sequences derived from the NADC30-like strains, a pattern also observed in this study. Given the crucial role of the NSP2 in PRRSV replication, virulence, and persistence [33], incorporation of the NADC30-like NSP2 region may facilitate immune evasion and contribute to prolonged viral persistence. This mechanism may partly account for the predominance of NADC30-like strains in recent PRRSV epidemics [34].

Under experimental conditions, the pathogenicity of different PRRSV strains in pigs varies markedly. In the pathogenicity test, piglets infected with GX-2428 developed more severe clinical symptoms, which may be associated with its unique genomic recombination pattern involving the ORF5 fragment derived from a QYYZ-like strain. Moreover, the GX-2428 group exhibited higher levels of inflammatory cytokines, suggesting that PRRSV infection induced an excessive inflammatory response. This response contributed to the development of clinical symptoms in PRRSV-infected piglets, including high fever and respiratory distress. Similar patterns have been observed in COVID-19 patients, where elevated levels of IL-4, IL-10, and TNF-α were detected in serum samples [35, 36]. Viremia and viral load in tissues are key indicators for monitoring PRRS disease progression [37, 38]. In this study, viremia was detected in all infected piglets at 3 dpi. By 21 dpi, viremia had become undetectable in the GX-3264 and GX-5430 groups, whereas it persisted at high levels in the GX-2428 group. However, tissue viral load analysis revealed that the GX-3264 and GX-5430 groups exhibited higher viral loads in the hilar lymph nodes, inguinal lymph nodes, and lungs. These findings demonstrate that during the persistent phase of PRRSV infection, viremia may become undetectable and clinical symptoms may subside, while the virus continues to reside in lymphoid tissues, thereby facilitating further transmission of PRRSV.

Interestingly, transcriptomic analysis revealed that the low-virulence strain GX-5430 induced a greater number of DEGs than the highly virulent strains GX-2428 and GX-3264. This finding contrasts with previous studies reporting that highly virulent PRRSV strains tend to induce a larger number of DEGs [39, 40]. This counterintuitive result may reflect distinct host–virus interaction strategies, whereby GX-5430, despite its lower pathogenicity, triggers broader transcriptomic reprogramming, potentially through mechanisms such as immune evasion, delayed immune activation, and interference with multiple metabolic and signaling pathways. Differences in host cell tropism, infection kinetics, and virus-induced cytopathology may also contribute to the variation in DEG profiles [41]. Moreover, viral loads in lung tissues varied among strains: GX-2428 exhibited substantial replication in the lungs, while GX-3264 and GX-5430 were primarily restricted to lymphoid tissues. These differences in tissue tropism may have contributed to the distinct transcriptomic profiles observed.

In the GX-2428 and GX-3264 groups, many upregulated genes were significantly enriched in innate immune pathways, including TLR signaling, NF-κB activation, and JAK–STAT signaling. These genes were also enriched in broader canonical immune pathways such as cytokine–cytokine receptor interaction and natural killer cell-mediated cytotoxicity [42]. These findings suggest that these two strains elicit robust innate immune and inflammatory responses, contributing to their relatively higher pathogenicity. In contrast, although GX-5430 infection resulted in the highest number of DEGs, it exhibited weaker enrichment in immune-related pathways. Instead, the DEGs were predominantly enriched in metabolic and signal transduction pathways such as Wnt, PI3K–Akt, and MAPK signaling pathways. Moreover, the upregulation of immune negative regulators such as *TNFAIP3* and *NFKBIA*, along with the significant downregulation of key antiviral genes such as *ISG15*, *IFIT3*, and *RSAD2*, were observed in the GX-5430 group [43, 44], further supporting the hypothesis that this strain suppresses host antiviral responses to facilitate persistent infection [45, 46].

In conclusion, this study successfully isolated and characterized three PRRSV strains from distinct lineages: GX-2428 (lineage 3), GX-3264 (lineage 1.8), and GX-5430 (lineage 1.5). Among them, GX-2428 and GX-3264 induced pronounced clinical symptoms in piglets, whereas GX-5430 resulted in a milder pathogenic profile. Despite its lower virulence and rapid clearance of viremia, GX-5430 maintained persistently high viral loads in certain tissues, highlighting the need for improved detection strategies for PRRSV. Furthermore, this study found that, in contrast to the lower pathogenicity of the GX-5430 strains, the highly virulent strains GX-2428 and GX-5430 cause tissue damage by inducing excessive inflammatory responses. These findings elucidated lineage-specific differences in virulence among PRRSV strains, thereby providing novel insights into PRRSV pathogenic mechanisms and offering a basis for improved prevention and control strategies.

## Supplementary Information

**Additional file 1.** **Primer sequence information for the RT-qPCR assay used in this study.**

**Additional file 2.** **Principal component analysis (PCA) and GO enrichment of DEGs based on RNA-seq data. A** PCA was performed to assess differences among samples. The x-axis and y-axis represent the first and second principal components, respectively. The samples closer together presented less variability. **B–D** GO enrichment analysis of DEGs in lung tissues from piglets infected with GX-2428 (**B**), GX-3264 (**C**), and GX-5430 (**D**), which were categorized into three major ontologies: classified into three major categories: Biological Process (BP), Molecular Function (MF), and Cellular Component (CC). The top 15 GO terms (or all if fewer than 15) in each category are shown. Red bars indicate upregulated genes, whereas blue bars indicate downregulated genes.

## Data Availability

The raw RNA-seq data were deposited in the NCBI Sequence Read Archive (SRA) under accession number PRJNA1280788.
