# Supplementary material for: Pathogenic and transcriptomic differences among porcine reproductive and respiratory syndrome viruses from distinct lineages in piglets
Source: Vet Res. 2025 Nov 28;56:224. doi: 10.1186/s13567-025-01659-w (PMC12664146; doi:10.1186/s13567-025-01659-w)
Supplement: Supplementary file 1 — Additional file 1. Primer sequence information for the RT-qPCR assay used in this study. [file 13567_2025_1659_MOESM1_ESM.docx]

**Additional file 1. Primer sequence information for the RT-qPCR assay used in this study.**

| Primers | Sequence (5’ to 3’) | Purpose |
| --- | --- | --- |
| ORF6-F | TTGCTAGGCCGCAAGTAC | Detect the viral load of PRRSV |
| ORF6-R | ACGCCGGACGACAAATGC |  |
| ORF6-Probe | FAM-CTGGCCCCTGCCCACCAC-TAMRA |  |
