# Supplementary material for: Pathogenic and transcriptomic differences among porcine reproductive and respiratory syndrome viruses from distinct lineages in piglets
Source: Vet Res. 2025 Nov 28;56:224. doi: 10.1186/s13567-025-01659-w (PMC12664146; doi:10.1186/s13567-025-01659-w)
Supplement: Supplementary file 2 — Additional file 2. Principal component analysis (PCA) and GO enrichment of DEGs based on RNA-seq data. A PCA was performed to assess differences among samples. The x-axis and y-axis represent the first and second principal components, respectively. The samples closer together presented less variability. B–D GO enrichment analysis of DEGs in lung tissues from piglets infected with GX-2428 (B), GX-3264 (C), and GX-5430 (D), which were categorized into three major ontologies: classified into three major categories: Biological Process (BP), Molecular Function (MF), and Cellular Component (CC). The top 15 GO terms (or all if fewer than 15) in each category are shown. Red bars indicate upregulated genes, whereas blue bars indicate downregulated genes. [file 13567_2025_1659_MOESM2_ESM.docx]

**
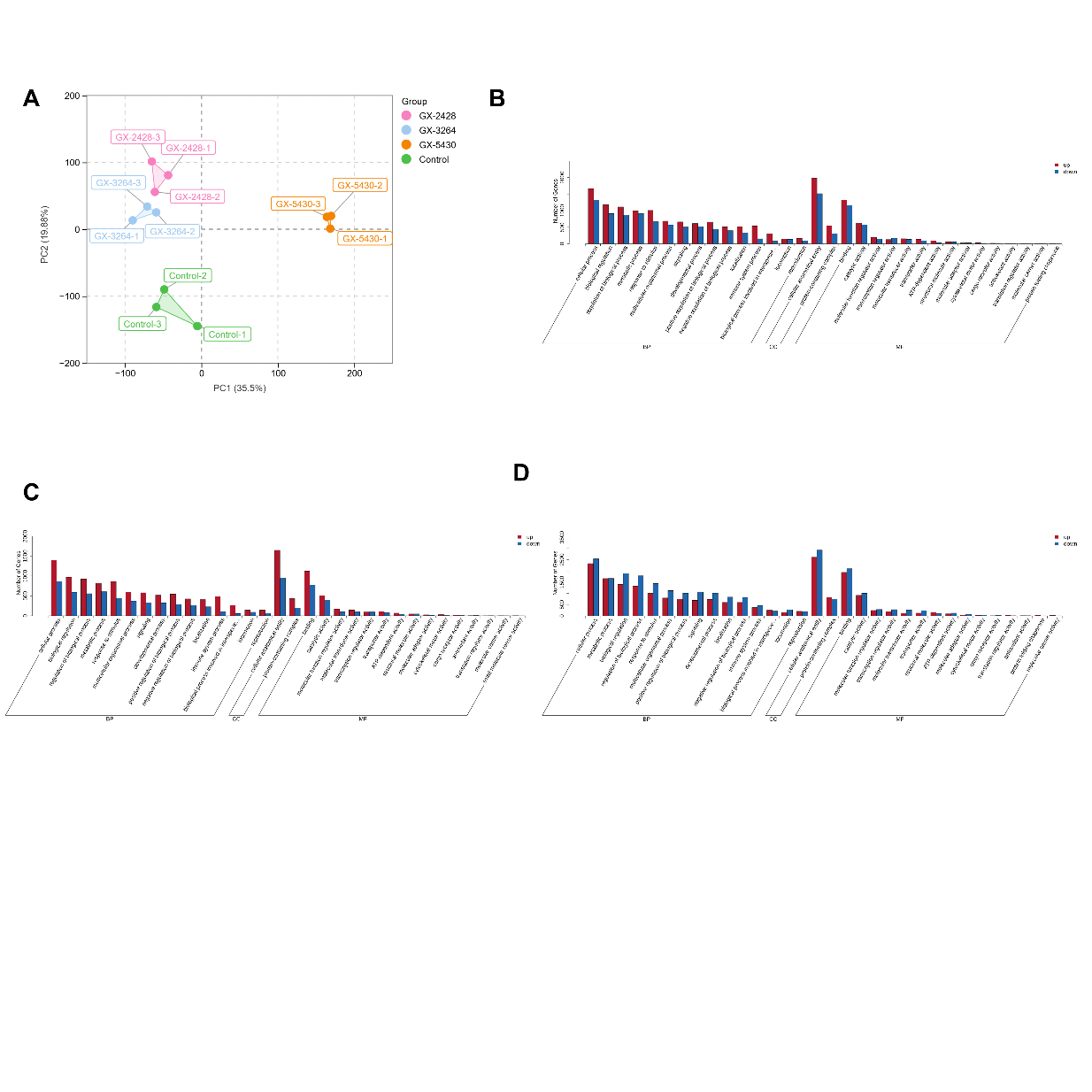
**

**Additional file 2. Principal component analysis (PCA) and GO enrichment of DEGs based on RNA-seq data.** **(A)** PCA was performed to assess differences among samples. The x-axis and y-axis represent the first and second principal components, respectively. Samples closer together exhibit less variability. **(B–D)** GO enrichment analysis of DEGs in lung tissues from piglets infected with GX-2428 **(B)**, GX-3264 **(C)**, and GX-5430 **(D)**, categorized into three major ontologies: classified into three major categories: Biological Process (BP), Molecular Function (MF), and Cellular Component (CC). The top 15 GO terms (or all if fewer than 15) in each category are shown. Red bars indicate upregulated genes, while blue bars indicate downregulated genes.
